# Supplementary material for: Cathepsin L Inhibitors with Activity against the Liver Fluke Identified From a Focus Library of Quinoxaline 1,4-di-N-Oxide Derivatives
Source: Molecules. 2019 Jun 26;24(13):2348. doi: 10.3390/molecules24132348 (PMC6651555; doi:10.3390/molecules24132348)
Supplement: Supplementary file 1 [file molecules-24-02348-s001.pdf]

Cathepsin L Inhibitors with Activity Against the Liver Fluke Identified From a Focus Library of Quinoxaline 1,4-di-*N*-Oxide Derivatives

Florencia Ferraro [1,2], Alicia Merlino [2,†], Jorge Gil [3], Hugo Cerecetto [4], Ileana Corvo [1,*] and Mauricio Cabrera [1,*]

[1] Laboratorio de I + D de Moléculas Bioactivas, Departamento de Ciencias Biológicas, CENUR Litoral Norte, Universidad de la República, Paysandú, 60000, Uruguay; fferraro@fcien.edu.uy

[2] Laboratorio de Química Teórica y Computacional, Instituto de Química Biológica, Facultad de Ciencias, Universidad de la República, Montevideo, 11400 Uruguay; amerlino@fcien.edu.uy

[3] Laboratorio de Reproducción Animal, Producción y Reproducción de Rumiantes, Departamento de Ciencias Biológicas, CENUR Litoral Norte-Facultad de Veterinaria, Universidad de la República, Paysandú 60000, Uruguay; jujogil@gmail.com

[4] Grupo de Química Medicinal, Laboratorio de Química Orgánica & Área de Radiofarmacia, Centro de Investigaciones Nucleares, Facultad de Ciencias, Universidad de la República, Montevideo, 11400, Uruguay; hcerecetto@cin.edu.uy

† Dedicated to the memory of Prof. Alicia Merlino, deceased 07/08/2018, colleague and friend.

* Correspondence: ilecorvo@gmail.com (I.C.), macabrera@fcien.edu.uy (M.C.); Tel.: +598-47227950-ext. 131 (I.C.); +598-47227950-ext. 131 (M.C.)

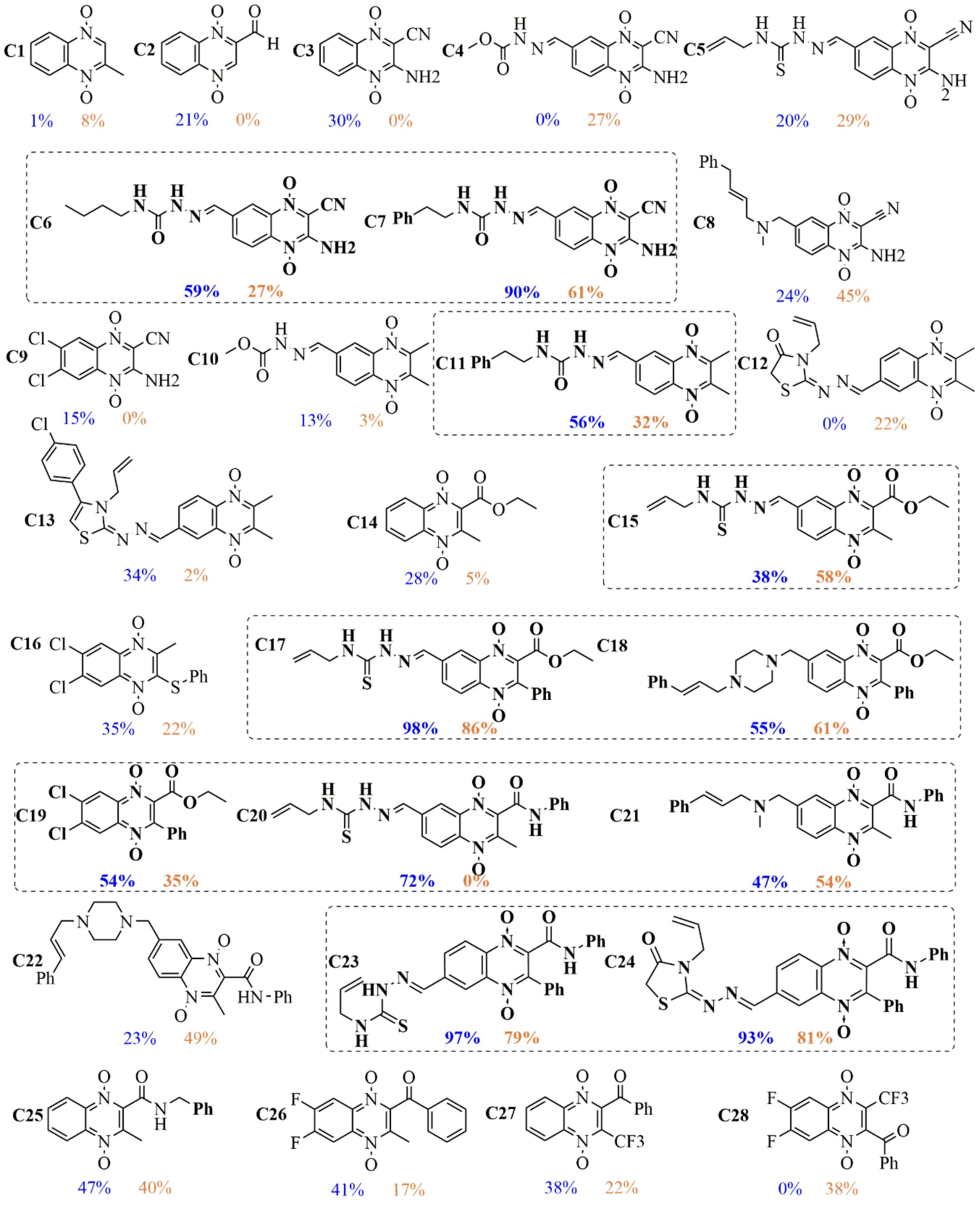

Figure S1. Structure of the 28 quinoxaline 1,4-di-*N*-oxide derivatives evaluated. In dashed boxes and bold are highlighted the eleven best inhibitor compounds. In blue and orange are shown the inhibition percentages for *Fh*CL1 and *Fh*CL3, respectively, at a 10 µM concentration.

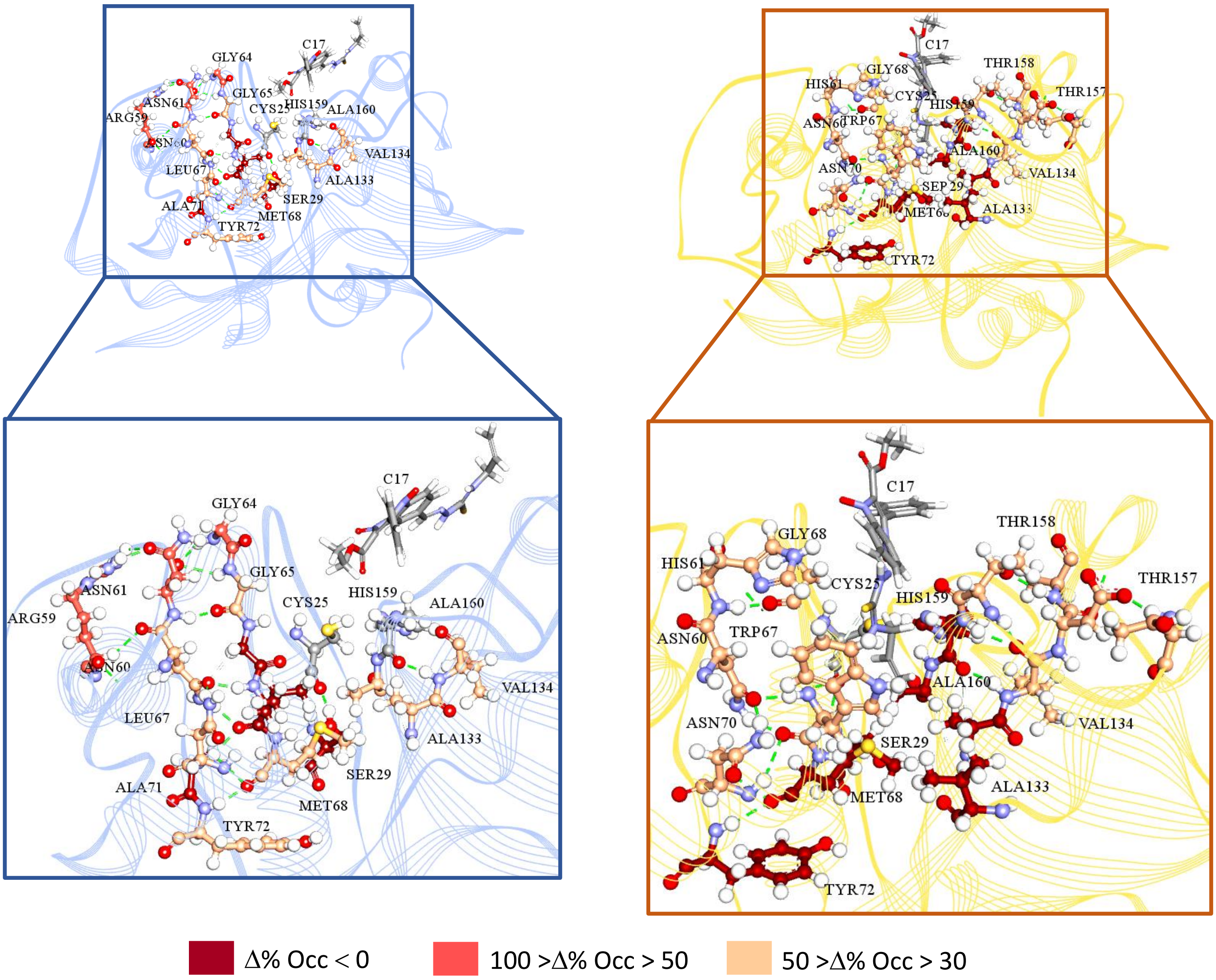

Figure S2. Hydrogen bonds varying in the presence or absence of **C17** bound to *Fh*CL1 (skyblue, left panel) and *Fh*CL3 (orange, right panel). Residues involved in hydrogen bonds (HB) are depicted in ball and sticks and coloured by ΔOcc (delta of occupancy) as indicated. ΔOcc is calculated with the formula ΔOcc=Occ*Fh*CL_**C17**—Occ*Fh*CL. HB are represented by green dashed lines and the catalytic Cys-His dyad is coloured by atom while **C17** is depicted in sticks.
